# Leo&Giulia: a cartoon series to promote health and prevention in primary school-aged children: Anna Odone

**DOI:** 10.1093/eurpub/ckac129.589

**Published:** 2022-10-25

**Authors:** R Vecchio, A Meloni, L Gentile, M Gaeta, C Signorelli, A Odone

**Affiliations:** 1 Department of Public Health, University of Pavia, Pavia, Italy; 2 School of Medicine, Vita-Salute San Raffaele University, Milan, Italy

## Abstract

**Background:**

Coronavirus (COVID-19) pandemic control measures actively involved people who were called to adopt new and unusual lifestyles. In this context, children had to stay home from school for weeks, had to adapt to new teaching methods and give up socializing. In many countries, not much attention was paid to the educational sector, and, ultimately, to children's physical and psychological well-being.

**Objectives:**

In this context, we developed an innovative health education audio-visual model to teach children about public health, empower them to adopt preventive behaviours and limit the risk of infection transmission in schools and in the community.

**Results:**

We designed and produced the animated cartoon series “Leo&Giulia” to convey solid scientific content and key public health messages related to the ongoing COVID-19 pandemic to primary school-aged children. Contents and dialogues were validated by a scientific committee composed of experts in the fields of public health, paediatrics, infectious diseases, and neuroscience, as well as communication experts. The first episode of Leo&Giulia focused on COVID-19 and explained to children what SARS-CoV2 was, its transmission and why schools were closed. Endorsed by the European Commission, it was broadcasted by national public and private television channels and went viral on social media. The second episode of Leo&Giulia, funded by the Italian Ministry of Research, was launched in April 2022 and focused on vaccines and immunization explaining to children how vaccines work and why herd immunity is important for collective health.

**Conclusions:**

Leo&Giulia is an innovative health education project to help children to better understand how to cope with COVID-19 as a public health challenge. More broadly, the series aims to increase youth engagement by promoting public health values and healthy behaviours.

**Key messages:**

• Health promotion targeting children is important and contributes to societal health and wellbeing.

• Cartoon series are an innovative digital health education tool that effectively increase youth engagement on public health values.

